# The Literary Digest

**Published:** 1895-04

**Authors:** 


THE LITERARY DIGEST. A repository of contemporaneous thought and research as presented in the periodical literature of the world in all departments of human life and action. Issued weekly; 30 quarto pages each week. Illustrated. Price, $3 per year. Funk & Wagnalls Co., Publishers, 30 Lafayette Place, New York.

Those who subscribe to this magazine will receive, as a premium, *The Hoyt-Ward Cyclopedia of Practical Quotations.* Prose and poetry, English and Latin, with an appendix containing proverbs from the Latin, French, German, and other modern foreign languages, all with their translations; law and ecclesiastical terms and significations; names, dates, and nationality of quoted authors, etc., with copious topical and other indices. By J. K. Hoyt and Anna L. Ward. Twenty thousand quotations—fifty thousand lines of concordance. A most valuable reference book. Royal 8vo, 907 pp. Price, tan sheep, $7.00.

